# TomoScore: A Neural Network Approach for Quality Assessment of Cellular cryo-ET

**DOI:** 10.21203/rs.3.rs-5405930/v1

**Published:** 2025-04-28

**Authors:** Xuqian Tan, Ethan Boniuk, Anisha Abraham, Xueting Zhou, Zhili Yu, Steven J. Ludtke, Zhao Wang

**Affiliations:** 1 Verna and Marrs McLean Department of Biochemistry and Molecular Pharmacology, Baylor College of Medicine, Houston, TX 77030, USA; 2 Bioengineering Program, Rice University, Houston, TX 77030, USA; 3 Chemistry and Sociology Program, Rice University, Houston, TX 77030, USA; 4 CryoEM Core (Advanced Technology Core), Baylor College of Medicine, Houston, TX 77030, USA; 5 Department of Molecular and Cellular Biology, Baylor College of Medicine, Houston, TX 77030, USA; 6 Department of Materials Science and Nanoengineering, Rice University, Houston, TX 77030, USA; 7 Department of Molecular and Cellular Oncology, Division of Basic Science, The University of Texas MD Anderson Cancer Center, Houston, TX 77030, USA

## Abstract

Electron cryo-tomography (cryo-ET) is a powerful imaging tool that allows three-dimensional visualization of subcellular and molecular architecture without chemical fixation. Tomogram quality varies widely, particularly during large high-throughput data collections, and the most common strategy for initial quality assessment is empirical judgment by an expert. Tomograms may be collected for two distinct purposes: annotation of subcellular features and cellular morphology, typically performed at lower magnifications and higher defocus, and subtomogram averaging, at high magnifications, closer to focus. For the first purpose, contrast and the ability to distinguish cellular features of interest are key, whereas for subtomogram averaging, recoverable signal at high resolution is the key factor. We have developed “TomoScore” a deep-learning based tomogram screening tool targeting cellular annotation. This tool provides a single quantitative measure of the suitability of a tomogram for annotation of subcellular features, in terms of the scale of features that can be readily distinguished. We further explore the relationship between accumulated electron dose and resulting quality, suggesting an optimum dose range for cryo-ET data collection. Overall, our study streamlines data processing and reduces the need for human involvement during pre-selection for tomogram segmentation.

## Background

Electron cryo-tomography (cryo-ET) is the only current technique capable of providing the 3-D structure of unlabeled/unfixed cells at nanometer resolutions^1,2^, as well as the emerging method of subtomogram averaging, providing high-resolution macromolecular structure *in-situ*^3^. These two related goals, subcellular annotation and subtomogram averaging, generally require different data collection parameters. For cellular annotation, lower magnifications provide larger fields of view, and higher defocus provides improved low-resolution contrast to reduce ambiguity when averaging is not being performed. For subtomogram averaging, higher magnification is required to achieve the necessary sampling, and lower defocuses are required to make accurate CTF correction possible at high resolution. For subtomogram averaging tools, such as CTFFIND^4^, can provide good estimates of whether tilt series contain enough high-resolution information to be suitable. However, for cellular annotation, CTF analysis is far less useful, as measurable Fourier contrast may be present even when tomograms lack sufficient features for annotation.

Accurate interpretation of cellular tomograms requires annotation of features at different levels of detail, ranging from individual macromolecules to organelles, depending on the purpose of the study^5–7^. However, tomogram quality can vary quite widely with imaging conditions and how the cells were prepared for imaging. For example, a thicker lamella provides a larger cellular volume but decreases the contrast of individual features within the cell. When a project involves hundreds to thousands of cellular tomograms, prescreening tomograms to assess suitability for annotation is critical. This process is often performed manually by a human visually screening and, in some cases, trying to annotate each tomogram in the set^8,9^. To reduce the need for this time-consuming process, we have developed a deep-learning based quantitative quality metric that can help rank tomograms, as well as assess approximate thickness. The “quality” we assess in this context is a measure of the smallest reliably resolvable feature present in individual 2-D slices of the tomogram. The quality metric is determined for all tomogram slices, providing an overall quality metric and thickness estimate. This metric is completely independent of typical resolution measures, such as the FSC between tomograms reconstructed from even movie frames and odd movie frames, which can produce both over and underestimates of the tomogram quality for annotation purposes^10–13^. Therefore, our metric only accesses the quality of the final cellular reconstructed tomograms.

Since the problem of scoring tomograms is mathematically ill-defined, we made use of deep learning techniques, building on ResNet^14^, with human-derived training data to produce our trained SliceQuality Network. The network was trained across multiple cellular species, microscopes, and instrument settings. The strategy we used for manually quantifying quality is straightforward and has minimal annotator-dependence.

## Results

### Human-assessed quality categories for network training

For model training, we needed to generate a training set with a wide variety of input images with corresponding human-judged categorical quality. To build a comprehensive training data set, we selected various prokaryotic and eukaryotic cells from the Cryo-ET Data Portal (Chan Zuckerberg Imaging Institute, Chan Zuckerberg Initiative)^15^. We also included platelet tomograms generated by our lab as a specialized type of eukaryotic cell (anucleate) due to their unique cellular structure (Supplement Table 1). Overall, 58,527 slices from 114 tomograms across 19 species were gathered and manually assessed.

**Figure 1.**
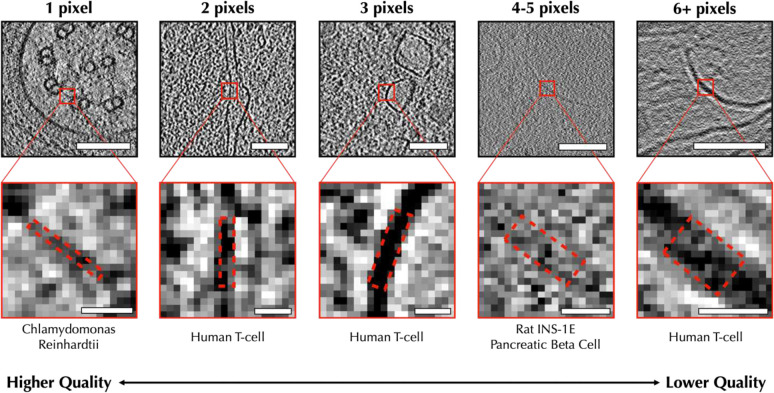
Demonstration of different quality tomogram slices under human-defined scoring criteria This figure demonstrates how membrane features are scored based on their size in pixels. Example features are included from C. reinhardtii (left), Human T-cells (mid-left, middle, right), and Rat INS-1E pancreatic beta cells(mid-right). The dashed red rectangles indicate the width measured for each feature. Images on the top row have been cropped to 200×200 pixels with a scale bar of 100 nm. Images on the bottom row have been cropped to 20×20 pixels with a scale bar of 10 nm. From left to right, the angstrom per pixel values of each image are 13.68, 18.92, 18.92, 14.08, and 9.867 Å/pix respectively. Additional examples taken from other species, as well as measurements for particle features, can be found in Supplementary Fig. 1.

Empirically, humans generally assess tomogram quality by examining 2D slices through the tomogram roughly paralleling the beam path of the specimen. While orthogonal slices can provide some additional insights, due to the missing wedge and variability between algorithms, these other views are less useful for assessing tomogram quality. Most segmentation/annotation algorithms use a similar approach of annotating 2D slices before merging the results into a 3D annotation. We followed a similar strategy for our human-based quality assessment, presenting one 2-D slice at a time for independent assessment. While the trained network produces a continuous quality variable, we limited human assessors to 6 discrete quality values based on the size in pixels of the smallest reliably discernible biological feature in each slice (Fig. 1). In addition to the 5 discrete feature-size categories presented in Fig. 1, a sixth category is reserved for tomogram slices with no discernible biological features at all.

During the human assessment of tomogram quality, we observed a logical trend: within one tomogram, central slices tend to have the highest quality, while the top and bottom slices have much lower quality. To encourage training the network to produce a smooth gradation between discrete scores, we applied a Gaussian filter to human-judged categories to generate continuous quality estimates across each tomogram.

We employed the following two strategies during data partitioning to establish rigorous training. Firstly, we performed a tomogram-based partition for unbiased training. Specifically, 98 of 114 tomograms were randomly selected for the training set pool, while the remaining tomograms were left for validation (1/114) and testing (15/114). This ensures the model does not peek into the testing tomogram due to highly similar neighboring slices from the same tomogram. Next, we performed a sample size partition for balanced training. Among the selected 98 tomograms, 2500 slices were randomly selected from each human labeled category to be added to the training, ensuring the prediction is not affected by sample size. Subsequently, our training set contains a total of 15,000 slices evenly distributed across six categories with corresponding continuous quality estimations.

### SliceQuality Network, a modified ResNet101 model, presents high accuracy for quality score prediction.

**Figure 2:**
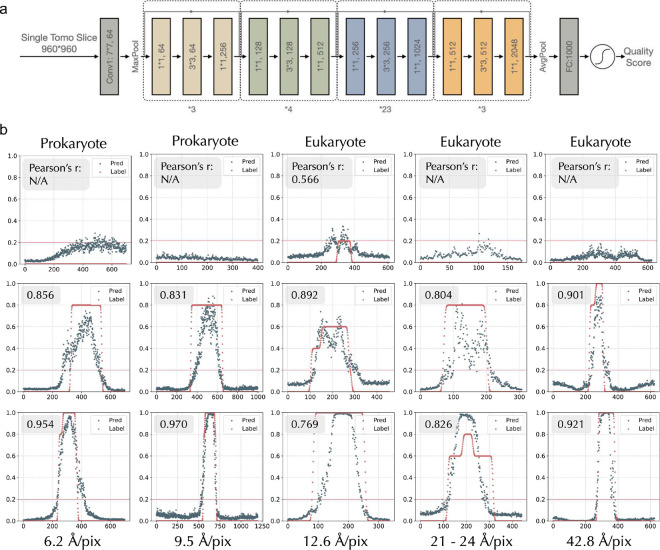
SliceQuality Network structure and training result a). SliceQuality Network is modified from ResNet101 structure. A sigmoid function is appended before the output layer to give a predicted quality score within the range 0–1. b). SliceQuality Network’s prediction score vs. label based on slice position curve for 15 test tomograms excluded from the training process. Pearson correlation coefficient is calculated (labeled on the top left) for each tomogram’s prediction-label pair, and all of them are statistically significant (p<0.001). Four of the lower-quality tomograms did not receive the Pearson correlation coefficient due to their constant labeling of 0. Each image has been cropped to 960×960 pixels for comparison. The first row captures the following, from left to right: *Sulfolobus solfataricus*, *Treponema primit*ia, human umbilical vein endothelial cell (HUVEC), human blood platelet, and human blood platelet. The middle row captures the following, from left to right: *S. solfataricus*, *Vibrio cholerae*, HUVEC, HUVEC, human blood platelet. The last row captures the following, from left to right: *Magnetospirillum magneticum*, *V. cholerae*, HUVEC, HUVEC, and human blood platelet.

We adopted the structure of ResNet101^14^ and modified it to predict the quality score of a single tomogram slice, as shown in Fig. 2a. Specifically, to obtain an output quality score ranging from 0 to 1, we added a sigmoid function at the end of ResNet101. Our continuous quality estimations in the training set uniformly map to the 0–1 prediction range (0.2: 6+ pixels; 0.4: 4–5 pixels; 0.6: 3 pixels; 0.8: 2 pixels; 1.0: 1 pixel). Our model takes less than 30 seconds to produce predictions for a standard 1k*1k tomogram containing ~200 slices on a machine with an NVIDIA GeForce RTX4090 GPU. Which is much faster than manual labeling.

To test model accuracy and generalizability, we selected 15 tomograms as the test set across different cellular species, microscopes, and magnifications. To quantitatively measure model accuracy, we overlaid the label scores and predictions and then plotted them by each slice’s position within one tomogram. An average Pearson correlation coefficient of 0.845 (p < 0.001) is reached between the prediction and label (Fig. 2b). Notably, our model also performs well on the three species (*Sulfolobus solfataricus*, *Treponema primitia*, *and Magnetospirillum magneticum*) that were not included in the training set, demonstrating its generalizability. We also noticed that two of the tomograms (row2, col2, and row3, col3) have a wider range of higher-quality slices for human labeling. After manual inspection, we found that both tomograms had tilted samples, resulting in the clear-cellular feature proportion of top and bottom slices being smaller than usual, thus receiving a lower predicted quality score. Further, our model also demonstrated its ability to differentiate between biological features and non-biological features, such as empty holes (row1, col3) and ice layers (row1, col4).

### SliceQuality Network accuracy and consistency were further validated by tomogram accumulated dose *in silicon* variations.

**Figure 3:**
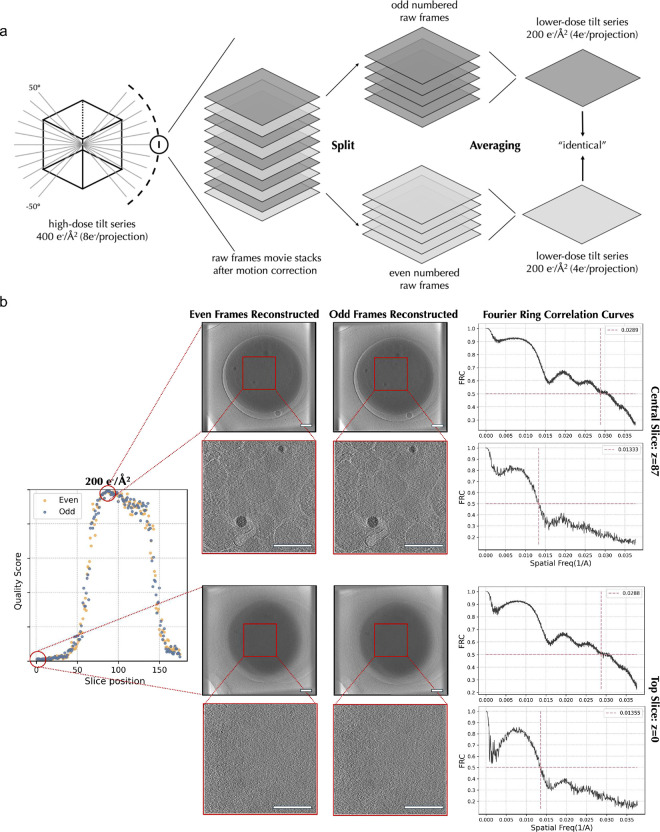

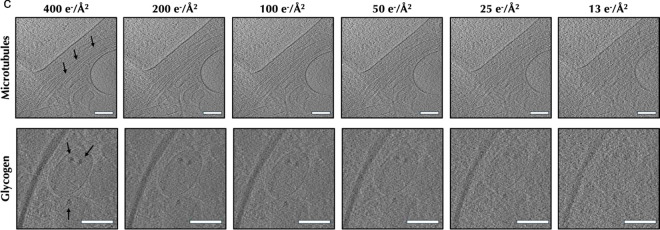
Model consistency tests on generated “identical” tomograms a) A workflow of separating even/odd raw frames from a single 2D tilt projection. Separated raw frames were averaged and merged into two parallel 2D tilt projections. Repeat this process for all tilt projections in a series to generate two identical sets of tilt series such that each set is exactly half dose of the original tilt series. Each set of half-dose tilts was then reconstructed into a 3D tomogram using the same alignment parameters. b) The even/odd frames reconstructed tomogram slices are similar by visual comparison. The Fourier ring correlation (FRC) curve also shows that both central and top slices from the two tomograms share high similarities at lower resolution frequencies. On the right, quality scores predicted by our trained model indicate that central slices have better resolvability (i.e higher quality score), where the FRC curves fail to show. c) Level of radiation exposure differentially affects tomogram quality. At higher radiation doses, tomograms of the same platelet exhibit higher-quality structures of microtubules and glycogen. At lower doses, tomograms exhibit more noise and structures are less defined. Scale bars are 200 mm

Assuming that our model is consistent and accurate, two identical tomograms should produce the same quality score using our model. However, practically, identical tomograms do not exist. Therefore, we developed an *in-silico* method to create “identical” tomograms to test model consistency and accuracy (Fig. 3a). First, we obtained a separate dataset containing 10 platelet tomograms with a high accumulated dose. Specifically, we recorded 152 raw frames per tilt image, with the total dose per tilt series being 400 e^−^/Å^2^. The movie frames from each angle were separated into two stacks based on their even or odd positions. These stacks are expected to contain identical low-frequency signals discernible to the human eye while exhibiting varied high-frequency noise. Consequently, we obtained two “identically resolved” tomograms (Fig. 3b) using the same reconstruction parameters. We calculated the Fourier Ring Correlation (FRC) curves between the even-frames and odd-frames reconstructed tomograms and noticed the central slice (higher-quality slice) and top slice (lower-quality slice) share a similar trend of FRC curves. Our model, on the other hand, can distinguish the quality differences clearly between the central and top slices.

We generated a series of tomograms of different accumulated doses (200 e^−^/Å^2^ to 13 e^−^/Å^2^) from the same tilt series by iteratively performing the even/odd splitting. Using our SliceQuality Network, we obtained the per-slice quality scores for the tomogram series with different accumulated doses, shown in (Supplement Fig. 2a). We further confirmed consistency between tomogram score predictions generated from even and odd frames at every accumulated dose level. Notably, all even/odd quality score differences were significantly lower than 16.7% (⅙), which is the discrete step size of the human categorization. This result indicates the prediction is on par with human judgments (Supplement Fig. 2b). Thus, we confirmed that our SliceQuality Network generates robust quality predictions at different doses.

Further, we observed that the predicted quality score peaks decrease as the accumulated dose decreases, indicating that there’s a decrease in overall tomogram quality along with a decrease in the simulated cumulative dose used in the reconstruction^16^. We performed a visual comparison at the same position in the tomogram to validate the resolvability of certain structural feature changes according to the dosage. We chose two specific positions within the tomograms that effectively encapsulate the key features considered in the initial ranking criteria. As shown in Fig. 3d, 400 e^−^/Å^2^, 200 e^−^/Å^2^, and 100 e^−^/Å^2^ tomograms clearly show microtubules with linear morphology and separation between neighboring objects to allow clear segmentation. Glycogens appear as high-contrast circular structures with precise boundaries to the cell environment in 400 e^−^/Å^2^ and 200 e^−^/Å^2^ tomograms. At lower dosages, organelles such as mitochondria lose contrast, which compromises the ability to visually identify the organelle by distinct features such as the double membrane and cristae. These results and observations inspired us to develop the following algorithm.

### A novel metric, TomoScore, was created for cryo-ET quality assessment.

As we previously defined, a predicted quality score of 0.2 maps to the threshold for the presence of biological features (6+ pixel size). We consider any slices scored above 0.2 to contain biological features. Therefore, we estimated the sample thickness by counting the number of slices scored above 0.2. We validated the estimated thickness with actual thickness measurements from YZ projection (Supplement Fig. 3a,b,c) on 137 tomograms (excluded from the training) through the RANSAC algorithm^17^ (Supplement Fig. 3d) and obtained a regression slope of 0.988 (p<0.001). We manually examined each outlier tomogram and concluded three reasons: 1) missing wedge between angle steps. This causes some tomograms to contain smearing structure features propagated through the z-axis after reconstruction, leading the model to make a thicker estimation; 2) tilted samples would also often make overestimation for thickness; and 3) low tomogram resolvability. The most recent method for sample thickness estimation by CTFFIND5^4^ makes estimations specifically for protein-level samples, while our estimation focuses more on cellular samples. Thus, this method was not included in our comparisons.


$${Thickness}_{\mathrm{est}}=\#(SliceQuality\;Network(slice)\;>\;0.2)$$

To simplify inter-tomogram quality comparison, we created a metric called TomoScore. TomoScore is generated from the predicted quality scores from a single tomogram and can be interpreted as the combination of all slice quality scores normalized by the estimated thickness of the specimen. As a result, TomoScore calculates the average quality score of slices within a single cell to represent the overall quality of a tomogram. Consequently, TomoScore has an expected range between 0 and 1, where 0 indicates no slice within the tomogram reveals cellular structure and 1 would denote a theoretically perfect tomogram with all slices revealing the optimal quality of organelle structures.


$$TomoScore=\frac{\;\mathrm{sum}(\mathrm{SliceQuality}\;\mathrm{Network}(\mathrm{slice})>0.2)}{{\mathrm{Thickness}}_{\mathrm{est}}}$$

**Figure 4:**
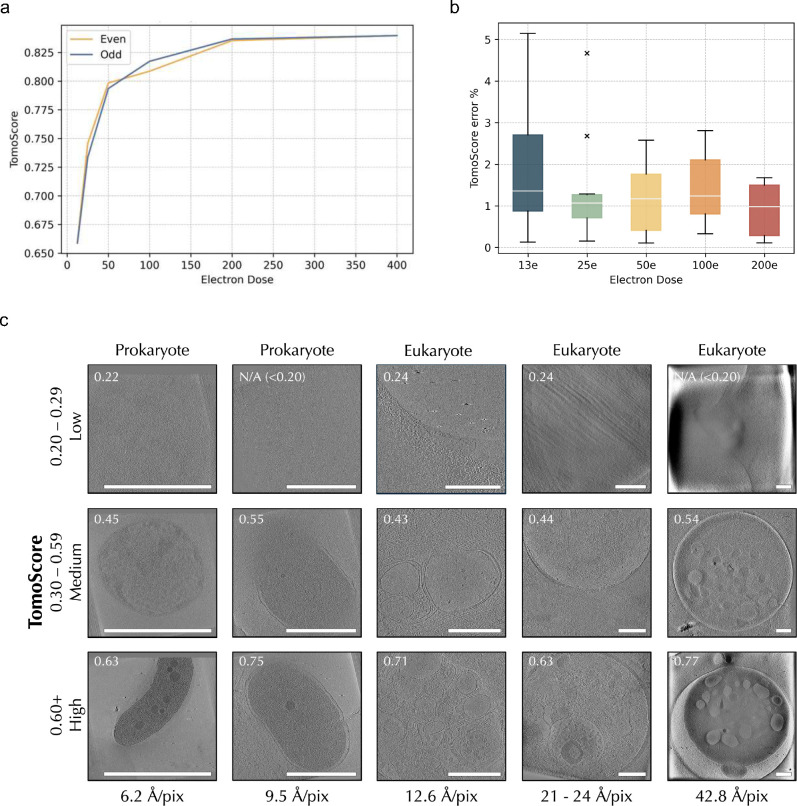
The trained model produces consistent TomoScore a) Calculated TomoScores for recursive even/odd dose splits of a sample high-dose tomogram. TomoScore decreases as the total doses decrease. b) TomoScore differences distribution for all 10 high-dose tomograms across different split dosages. c) TomoScore accurately predicts tomogram quality across various cell types and magnifications. We compare the most resolvable slice, as determined by a human, of the same 15 tomograms used for Fig. 2b. As predicted, tomograms with a high TomoScore are of better quality and have more resolvable subcellular structures, and all scale bars are 500 nm.

We further validated our model’s consistency on TomoScore using the same even/odd frames split tomograms. For a single set of tomograms split into different total doses, the TomoScore differences between even/odd tomograms are small across the total dose from 13 e^−^/Å^2^ to 200 e^−^/Å^2^ (Fig. 4a). We also have a small range of distribution for TomoScore differences between the 10 even/odd split tomograms across different total doses (Fig. 4b).

To verify TomoScore aligns with human quality assessment, we visually examined the same 15 tomograms from the test set. Fig. 4c demonstrates how higher TomoScores correspond to higher quality tomogram slices as perceived by the human eye. This trend is preserved between different cell types and magnifications, indicating the broad utility of TomoScore in predicting tomogram quality.

### TomoScore identifies the optimum electron dose range.

Calculating TomoScore for one of the tomograms separated into a series of different total doses, Fig. 4a also demonstrated a trend of increasing TomoScore with increasing simulated accumulated dose, which is consistent with common expectations^2^. Specifically, the TomoScore increases drastically in the lower total dose range (13 e^−^/Å^2^ to 100 e^−^/Å^2^) and reaches a plateau in the higher total dose range (100 e^−^/Å^2^ to 400 e^−^/Å^2^).

**Figure 5:**
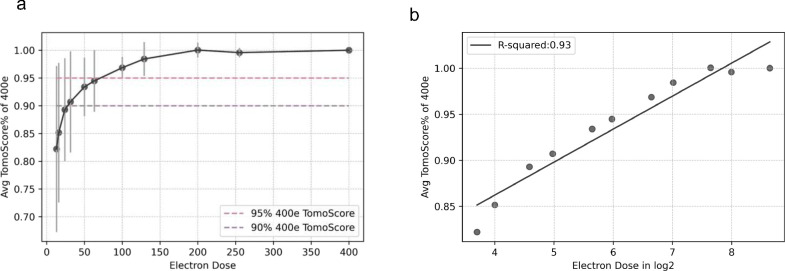
TomoScore determines the optimum dose range for cryo-ET data collection For each total dose, we calculated the percentage of TomoScore each split tomogram can reach its full-dose and plot the percentage distribution of all 10 tomograms with the dose range in a) linear scale and in b) log2 scale.

To further confirm that this is not a coincidental trend limited to a single tomogram, and to understand the correlation between TomoScore and accumulated doses, we applied similar tests to the previously mentioned 10 high-electron-dose tomograms and created 5 more accumulated doses tomograms (256 e^−^/Å^2^, 128 e^−^/Å^2^, 64 e^−^/Å^2^, 32 e^−^/Å^2^, 16 e^−^/Å^2^) by extracting different percentages of evenly spaced raw frames. We calculated and plotted the percentage TomoScore each split dose tomogram (13 e^−^/Å^2^, 16 e^−^/Å^2^, 24 e^−^/Å^2^, 32 e^−^/Å^2^, 50 e^−^/Å^2^, 64 e^−^/Å^2^, 100 e^−^/Å^2^, 128 e^−^/Å^2^, 200 e^−^/Å^2^, 256 e^−^/Å^2^) can reach of its full dose tomogram (400 e^−^/Å^2^) in Fig. 5a. In each set of tomograms, we observed the same trend that TomoScore is positively correlated with the total dose. At a dose range between 80–120 e^−^/Å^2^, the tomograms achieved approximately 95% of their respective maximum score. Under a 60e-80e dose range, the tomograms attained around 90% of the highest score. In other words, data collection with an accumulated dose of 60–120 e^−^/Å^2^ is optimum for high-quality platelet tomograms.

Next, we converted the total dose axis into a log2 scale and replotted the average max TomoScore percentage for the 10 series of tomograms to better quantitatively characterize the trend between TomoScore and the total dose observed (Fig. 5b). Calculating the slope and intercept of the best-fit line for each tomogram series within this logarithmic scale, we observed a nearly perfect regression line (R^2^ = 0.93, p-value = 2.011*10^−6^). This result provides additional confirmation that all 10 tomograms exhibit a similar trend, where their TomoScore proportionally increases with the log2 of the total dose.

It is acknowledged that our method of simulating different accumulated doses takes less consideration of dose damage. Still, the 10 tomograms we used for dose simulation were acquired and processed using identical protocols, controlling for all other factors that could potentially influence quality disparities except for inherent cell-to-cell differences.

## Discussion

The application of deep learning techniques has opened new avenues for addressing the challenges of tomogram data processing. Our TomoScore quality evaluation system is especially useful for cryo-ET, where the datasets are large, complex, and noisy. New data collection strategies, such as Parallel/montage cryo-ET^18–21^ on Thermo Scientific EPU Software or similar SerialEM methods can collect multiple locations in one single angular step, increasing the throughput of cryo-ET data collection compared to traditional collection schemes. Moreover, rapid tilt-series acquisition^22^ or newer continuous tilt methods can acquire a complete tilt series in ~2 minutes, which increases CryoET collection speeds even further. Batch tomogram^23–25^ and most tomogram analysis software packages like IMOD^26,27^, EMAN2^28,29^, Tomoauto^30^, emClarity^31^, Aretomo^32^, and Warp^33^ can run automated or semi-automated reconstruction of tomograms in a pipeline. These improvements have dramatically increased the throughput of cryo-ET data collection and reconstruction. Therefore, an automated quality assessment tool specific to cellular cryo-ET is a critical step in high-throughput cryo-ET analysis pipelines.

In summary, our SliceQuality Network has proven capable of per-slice quality analysis of cellular cryo-ET across different species and magnifications. The resulting tool demonstrates the ability to accurately, consistently, and, more importantly, quickly assess the resolvability and thickness of reconstructed tomograms. TomoScore thus permits a large set of tomograms resulting from a data collection to be quality-ranked in order for further analysis. TomoScore is on par with human judgment and is fully automated, saving human effort for downstream processing.

Using TomoScore, we identified the optimal dose range for platelets under a specific set of experimental conditions. Clearly, other variables play a role in this optimal dose, such as properties of the cell type, selected defocus range, lamella thickness, and other issues, but for a given set of conditions, TomoScore does provide a method for optimizing the targeted dose^34–37^. By using an *in-silico* approach to control the accumulated dose, the resulting resolvability differences across different doses are not considered affected by radiation damage.

Overall, our SliceQuality Network, along with TomoScore can provide an overall estimation of the quality of cellular cryo-ET, taking all possible influencers into account. We successfully demonstrated this method is robust for tomogram quality prediction with high accuracy and consistency. We are confident that our TomoScore can contribute to quality standardization in the cryo-ET community.

## Methods

### Platelet sample preparation and freezing.

Freshly drawn healthy human blood (Gulf Coast Regional Blood Center) was centrifuged at 200G to obtain platelet-rich plasma (PRP), and further centrifuged at 100G to remove the residual red blood cells. We applied 2–4μL PRP to glow-discharged Quantifoil grids and used a Vitrobot (Mark IV, FEI Corp) to quickly freeze samples to form vitreous ice. Mouse platelet samples were taken from previous research^7^.

### Platelet data collection, processing and reconstruction.

Platelet data was collected using a 300 kV FEI Titan Krios microscope with a Gatan K2 Summit direct electron detector camera, through the SerialEM software. The magnification was 11,500x, with a pixel size of 13.26 Å. The tilt series were collected using a 2° angular step, unidirectionally from −50° to +50°. The defocus ranges from −10 to −15 μm. For the normal dose tilt series, the total dose was 100 e^−^/Å^2^. For the high-dose tile series, the total dose was 400 e^−^/Å^2^, achieved through increasing the exposure time of each tilt image. Each 400 e^−^/Å^2^ total dose tomogram’s micrograph was collected in 152 frames (0.052 e^−^/Å^2^/frame). All frames were corrected using MotionCor2^38^ with default setting to avoid beam-induced motion along with the gain correction.

Platelet tomograms used for model training were reconstructed using the automated pipeline in EMAN2^39^. The percentage of tilt images kept for reconstruction was 90%. The tomograms were initially output as bin2 tomograms, then another bin2 was applied by EMAN2.

For the high dose electron tomograms used for even/odd raw frames split, reconstruction was first done through EMAN2 with 100% tilt images kept on the original 400 e^−^/Å^2^ tomograms with full raw frames, and the alignment parameters were saved for later reduced dose reconstruction. The same alignment parameters were then imported with no alignment and 0 rounds of iteration using all tilt images for reduced-dose tomogram reconstruction to ensure consistency.

### Collect data from the Cryo-ET Data Portal.

To build our dataset for training, we used API from the Cryo-ET Data Portal (Chan Zuckerberg Imaging Institute, Chan Zuckerberg Initiative)^15^. Specifically, searched for tomograms of sizes larger than 800*800 pixels and of magnifications smaller than 50 Å/pixel but larger than 5 Å/pixel. We randomly picked 15 tomograms from each eukaryotic species and 3 tomograms from prokaryotic species that fit the selection criteria. Tomograms containing no visible biological features were excluded from our dataset after the first round of human screening. The remaining tomograms were added to our curated training dataset, as shown in Supplement Table 1.

To generate Fig. 4c, we selectively chose both prokaryotic and eukaryotic tomograms from the CryoET Data and blood platelet tomograms generated by our lab. We limited our selection to tomograms that had an Å/pixel value of 5 or higher to match the magnification range of the tomograms used in our training set. For each magnification level, we selected three tomograms that, to the human eye, had obvious differences in quality and ran TomoScore to confirm these differences.

### Human quality scoring criteria.

Human scoring was performed using the EMAN2^39^ 2D slice display function and its included measurement tools. Each slice of a tomogram was evaluated by the eye to find the smallest recognizable feature, and then the width of that feature in pixels would be measured and recorded. Pixel measurements within a range of ~1 pixel were treated as the same measurement to reduce human error, forming five discrete categories. Features from lower-quality tomograms were more blurry and less consistent, so there was a larger variation in pixel measurements for large features. To account for the inconsistency, the ranges of categories 1 and 2 were extended to >1 pixel. A sixth category labeled “0” was included as a measurement for slices in which no features were visible.

### Sample thickness measurement

When measuring the thickness of cells using a tomogram reconstruction, we took a 2D projection along the XZ or YZ planes of the tomogram and measured the bounds of the cell by hand along the vertical Z axis (Supplementary Fig. 3a). We chose to use the YZ slices for taking measurements since the missing wedge effect leaves large artifacts along the XZ slices. Using EMAN2, we averaged 513 adjacent YZ slices to generate a 2D projection along the X-axis, smoothing out the boundaries of the cell. Measurements for cell thickness were recorded as the distance in pixels between the lower and higher cell boundaries. To make sure our measurements matched the cell boundary, we examined the Z slices we chose as the bounds for the measurement (Supplementary Fig. 3bc). Measurement bounds were reasonable if the cell was visible in the slice, but no features were visible, and unreasonable if the cell was not visible at all.

### Data preprocessing and augmentation.

All tomogram slices used for this research were cropped into 960*960 in pixel size and then normalized using EMAN2 before any further analysis or training.

Due to the scarcity of slices with the smallest features of 4–5 pixels and 6+ pixels, rotation augmentation was applied to these slices such that slices of 6+ pixels were rotated 90 degrees 3 times (90°, 180°, 270°) and slices of 4–5 pixels were rotated 90 degrees 2 times (90°, 180°).

**Table:** 

Feature Diameter	No Feature	6+ pixels	4–5 pixels	3 pixels	2 pixels	1 pixel
#Slices after augmentation	33,580	2,920	3,410	4,147	5,285	4,963

Gaussian filter (σ=3, and z as the coordinate of the slice) was applied on human-judged ranks to generate continuous quality estimates across each tomogram.


$$g(z)=\frac{1}{\sqrt{2\pi }\sigma }e^{-z^{2}/\left(2\sigma^{2}\right)}$$

### SliceQuality Network training.

To achieve better generalization to ameliorate overfitting, we adopted the pre-trained weights of ResNet101 from PyTorch^40^. An adagrad variant of stochastic gradient descent was used accompanied by mean square error loss implemented in the PyTorch with a learning rate of 5*10^−7^ for 200 epochs. Each slice of tomogram put into training was of size 960*960 pixels, and the minibatch size was 8. The model was trained on a single NVIDIA GeForce RTX 4090 GPU of 24GB of VRAM, and the model took around 72 hours to train.

## Supplementary Material

Supplement 1
