# Supplementary material for: TomoScore: A Neural Network Approach for Quality Assessment of Cellular cryo-ET
Source: Res Sq. 2025 Apr 28:rs.3.rs-5405930. Preprint. [Version 1] doi: 10.21203/rs.3.rs-5405930/v1 (PMC12060984; doi:10.21203/rs.3.rs-5405930/v1)
Supplement: Supplement 1 [file NIHPPrs5405930v1-supplement-1.pdf]

Supplementary information

	Species	#Tomograms	#Slices
**Eukaryote**	Chlamydomonas reinhardtii	10	4616
	Homo sapiens	2	981
	Homo sapiens (T-cell)	13	3632
	Homo sapiens (HUVEC)	5	2150
	Schizosaccharomyces pombe	8	4972
	Mus musculus	5	1419
	Rattus rattus (pancreatic beta cells)	10	6078
**Prokaryote**	Bacillus subtilis	3	1126
	Escherichia coli	2	1791
	Helicobacter pylori	2	1293
	Legionella pneumophila	3	1489
	Helicobacter hepaticus	3	2388
	Magnetospirillum magneticum	1	700
	Nitrosopumilus maritimus	3	1488
	Pseudomonas aeruginosa	3	1789
	Spiroplasma melliferum	1	157
	Streptococcus pneumoniae	3	2092
	Sulfolobus acidocaldarius	3	2091
	Sulfolobus solfataricus	2	1400
	Treponema primitia	1	400
	Vibrio cholerae	5	4587
**Anucleate Eukaryote (Platelets)**	Homo sapiens	9	3395
	Mus musculus	17	8492
**Total**	**19**	**114**	**58527**

Table 1 Summary of tomogram dataset

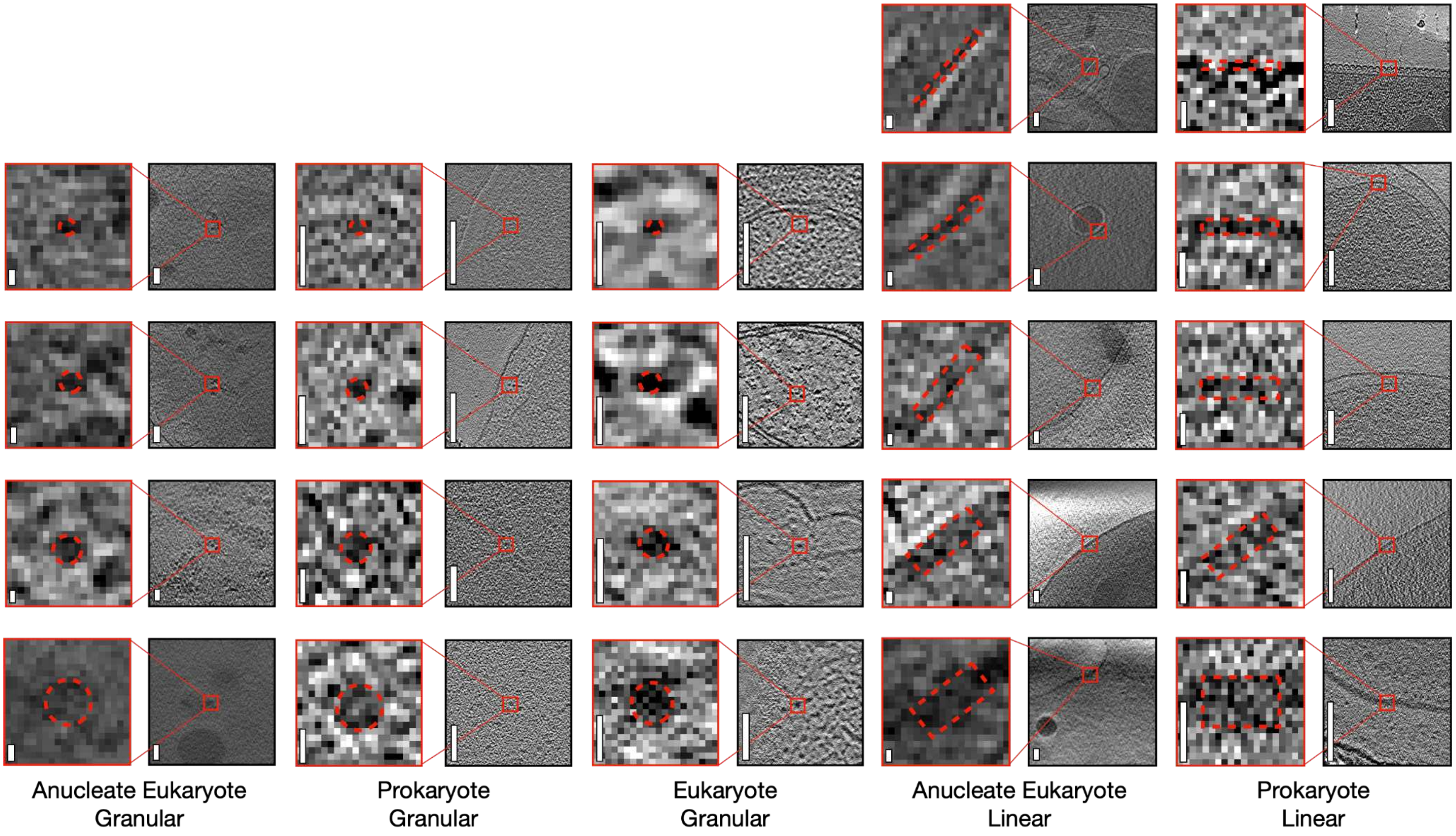
Anucleate Eukaryote Granular
Prokaryote Granular
Eukaryote Granular
Anucleate Eukaryote Linear
Prokaryote Linear

Supplementary Figure 1 Tomogram slices with quality-determining features

Additional examples of feature measurements in Eukaryotes (top rows), Prokaryotes (middle rows), and Anucleate Eukaryotes (bottom rows). Features are separated into membrane structures (left group), and particle structures (right group). Membrane features are measured by their width, while particle features are measured by their diameter. Black-bounded images are cropped to 200x200 pixels and have a scale bar of 100 nm. Red-bounded images are cropped to 20x20 pixels and have a scale bar of 10 nm.

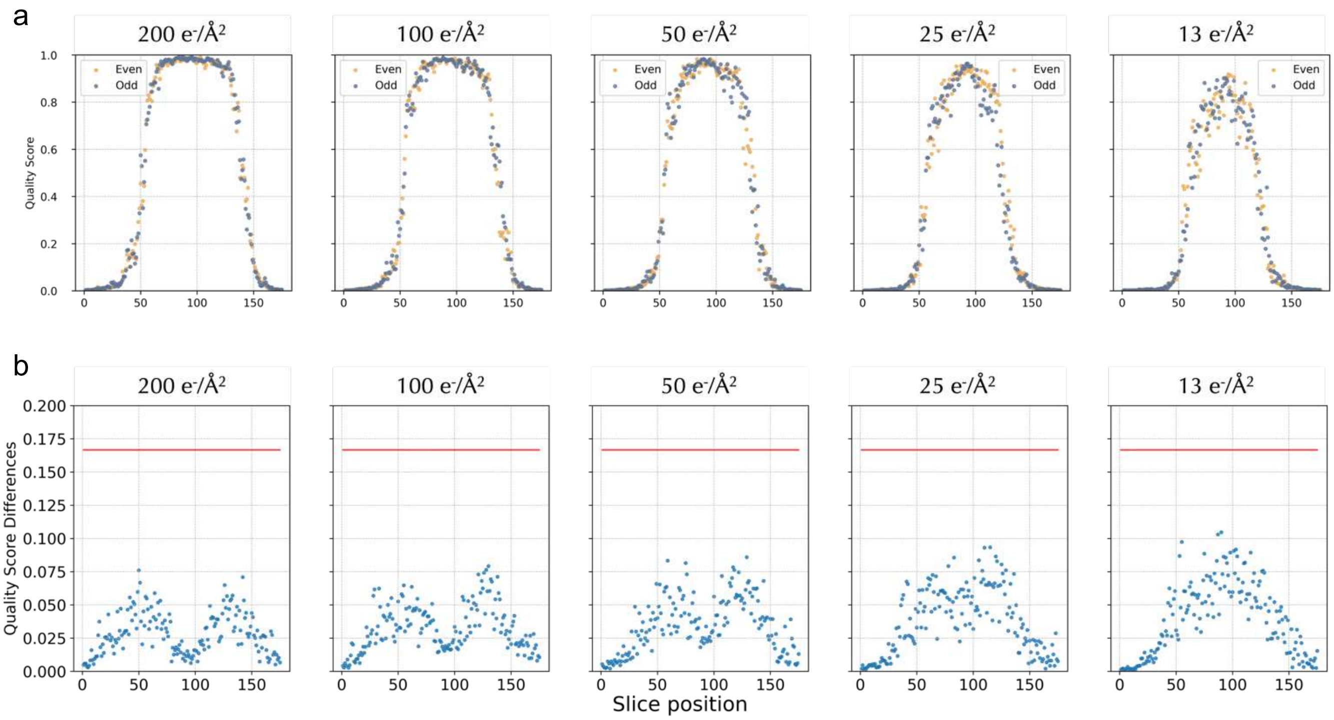

Supplementary Figure 2 Even/Odd frames reconstructed tomograms are “identical”

a) Shows the SliceQuality Network’s predictions for the same set of tomograms, where the quality scores predicted for even/odd splits of different decreasing total dosages. b) Average quality score differences of 10 tomograms split into different dosages vary within a small range, indicating the high consistency of our trained model.

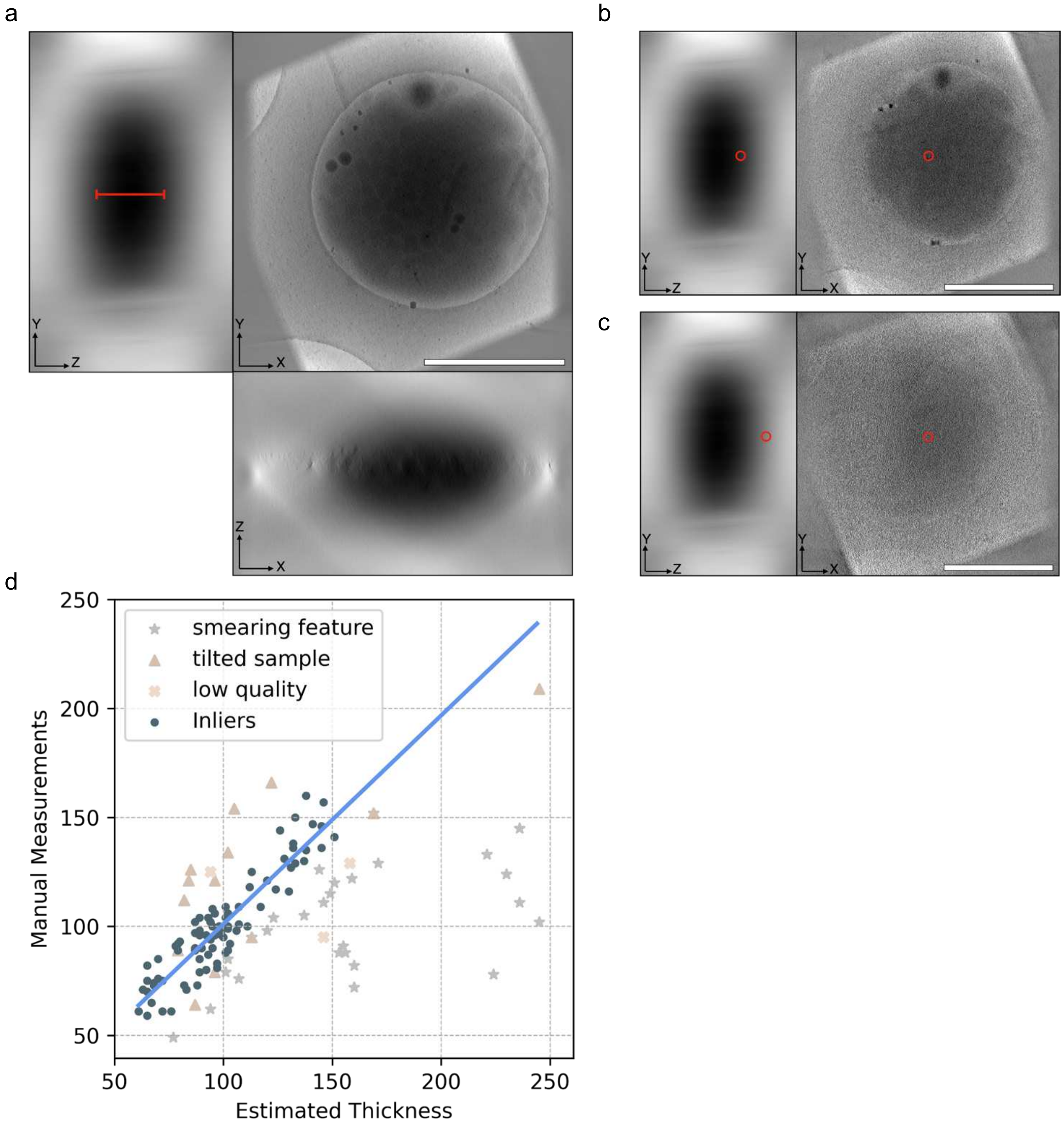

Supplementary Figure 3 Sample thickness estimation and validation

a) YZ, XY, and XZ projections of a platelet tomogram. The red bar shows which part of the image was used to measure cell thickness. b) The red circle indicates a point on the measured edge of the cell on the YZ projection. c) The red circle indicates a suggested alternative point of measurement taken at a lower contrast boundary. All scale bars are 1000 nm. d) RANSAC regression pinpointed inliers and outliers of the thickness estimations. 137 tomograms tested for thickness were all platelet cells. Reconstruction thickness varied from 175 to 600 slices in the z-direction.
